# Phytic acid as alternative setting retarder enhanced biological performance of dicalcium phosphate cement *in vitro*

**DOI:** 10.1038/s41598-017-00731-6

**Published:** 2017-04-03

**Authors:** Susanne Meininger, Carina Blum, Martha Schamel, Jake E. Barralet, Anita Ignatius, Uwe Gbureck

**Affiliations:** 1 Department for Functional Materials in Medicine and Dentistry, University of Würzburg, Pleicherwall 2, D-97070 Würzburg, Germany; 2 Department of Surgery, Faculty of Medicine, Faculty of Dentistry, McGill University, Montreal, Quebec H3A 2B2 Canada; 3 Centre for Musculoskeletal Research, Institute for Orthopaedic Research and Biomechanics, University of Ulm, Helmholtzstrasse 14, D-89081 Ulm, Germany

## Abstract

Dicalcium phosphate cement preparation requires the addition of setting retarders to meet clinical requirements regarding handling time and processability. Previous studies have focused on the influence of different setting modifiers on material properties such as mechanical performance or injectability, while ignoring their influence on biological cement properties as they are used in low concentrations in the cement pastes and the occurrence of most compounds in human tissues. Here, analyses of both material and biological behavior were carried out on samples with common setting retardants (citric acid, sodium pyrophosphate, sulfuric acid) and novel (phytic acid). Cytocompatibility was evaluated by *in vitro* tests with osteoblastic (hFOB 1.19) and osteoclastic (RAW 264.7) cells. We found cytocompatibility was better for sodium pyrophosphate and phytic acid with a three-fold cell metabolic activity by WST-1 test, whereas samples set with citric acid showed reduced cell number as well as cell activity. The compressive strength (CS) of cements formed with phytic acid (CS = 13 MPa) were nearly equal to those formed with citric acid (CS = 15 MPa) and approximately threefold higher than for other setting retardants. Due to a proven cytocompatibility and high mechanical strength, phytic acid seems to be a candidate replacement setting retardant for dicalcium phosphate cements.

## Introduction

Trauma or disease can cause bone defects exceeding a critical size such that bone grafts have to be implanted into the defect to fill space and to prevent fibrous tissue ingrowth during healing. The development of synthetic bone graft substitutes provides a wide range of possibilities to treat these defects. Major advantages of such synthetic materials compared to autologous transplants are their unlimited availability, constant quality and the absence of donor related pathogen transfer compared with natural resources^1, 2^. These synthetic materials can be either of an organic nature such as polylactid-co-glycolic acid^3, 4^ or they can be based on inorganic calcium phosphate chemistry to mimic the natural chemical composition of the bone mineral phase. Calcium phosphates are applied as either pre-formed monoliths or granules composed of sintered hydroxyapatite or tricalcium phosphate (or mixtures of both) or they can be used as self-setting cement pastes which can be molded or even injected^5, 6^ into a defect following the formation of a mechanically stable implant directly at the application site. Currently, calcium phosphate cements (CPC) are clinically used to fill and restore cancellous bone, to fix implants and to reinforce osteoporotic bone^7^. CPC inhibit fibrous tissue ingrowth^8^ and may have the potential to increase the mechanical strength of bone enabling treatments with screws for fixation^9–11^.

The setting mechanism of CPC occurs by a continuous dissolution/precipitation reaction based on the differential of solubilities of cement raw materials and their setting product. Mineral phases formed during cement setting are either stoichiometric or calcium deficient hydroxyapatite at neutral or basic pH conditions^12^, while secondary protonated dicalcium phosphates such as brushite (CaHPO_4_·2H_2_O)^13, 14^ or monetite (CaHPO_4_)^15, 16^ will be formed at an acidic pH. The latter have the advantage of a higher solubility under *in vivo* conditions compared with apatitic cements^17, 18^ (solubility of brushite (monetite) in water: 85–88 (41–48) mg/L, HA: 0.2 mg/L)^19^. A couple of studies have demonstrated the bone remodelling capacity of brushite forming cements in various animal models within a time period of 16–18 weeks^20, 21^, whereas other studies have indicated a phase conversion of brushite into lower soluble octacalcium phosphate or hydroxyapatite *in vivo* ^22^, which slows down degradation compared to monetite.

Since the crystallization rate of brushite crystals is approximately three orders of magnitude higher than those for hydroxyapatite^23^, setting retarders have to be used in these cements to adjust clinically appropriate setting times of 3–5 min. Most commonly applied setting retarders for brushite cements are citric acid^24, 25^, pyrophosphate ions^24, 26^, sulfate ions^27^ or magnesium ions^28, 29^. Among those, citric acid was shown to result in superior physical properties such as mechanical strength, setting time and injectability^30, 31^. Although all of these retarders were compounds naturally occurring in tissues^32^, the biological properties of brushite cements were recently demonstrated to be influenced by these additives. Jamshidi *et al*. showed that the cell attachment to brushite cements is mostly inhibited by using citric acid as retarder^33^. This was attributed to the formation of an amorphous dicalcium citrate phase during setting, which slowly dissolved afterwards in the culture medium during cell experiments. Another study by Kanter *et al*.^17^ revealed that citric acid may also influence the degradation rate of brushite cement *in vivo*. Even those cement samples, which were not converted into lower solubility octacalcium phosphate remained mostly stable during 10 months of orthotopic implantation, either because the calcium citrate complexes formed in the cement decreased the chemical solubility of the cement matrix or they decreased the metabolic activity of bone resorbing osteoclasts near the implant.

This study aimed to investigate this observation in more detail to identify the effect of different setting retarders on both basic properties of cements as well as on their influence on the cytocompatibility against bone forming and degrading cells. Several possible alternatives to citric acid as setting retarder in brushite cements such as sodium pyrophosphate, sulfuric acid and phytic acid were investigated and compared with citric acid and cements without any setting retardant. Apart from analyzing the influence of these additives on the physico-chemical cement properties such as initial setting time, compressive strength and the exothermic setting reaction, biological investigations concerning cytocompatibility were carried out with a human osteoblastic cell line (hFOB 1.19) and a murine macrophage cell line (RAW 264.7) differentiating into osteoclasts.

## Results

**Figure 1:**
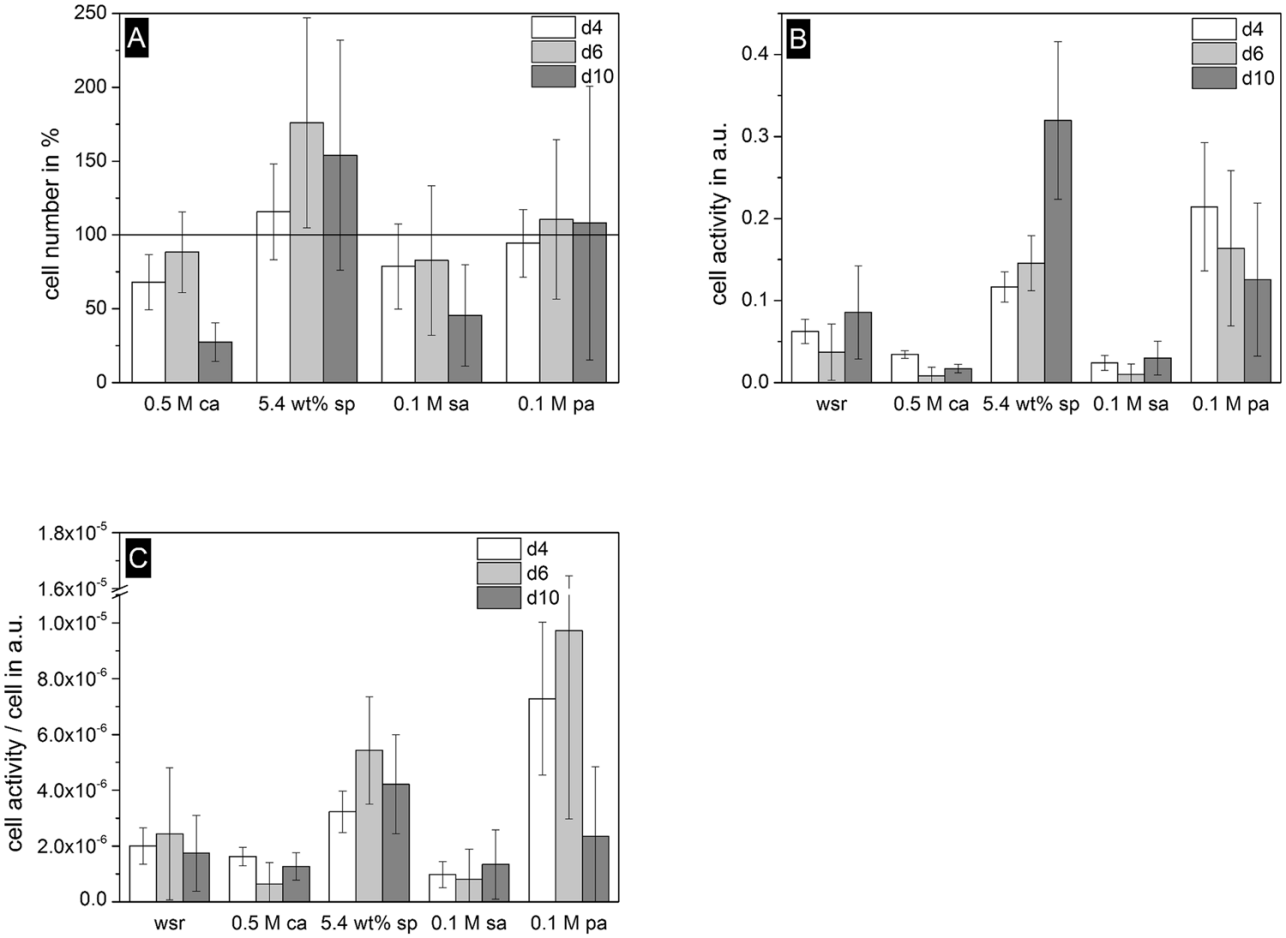
Percent cell number with regard to cement without setting retardant (solid line at 100%) (**A**), cell activity (**B**) and cell activity per cell (**C**) of hFOB. Samples without setting retardant (wsr) and retardants citric acid (0.5 M ca), sodium pyrophosphate (5.4 wt% sp), sulfuric acid (0.1 M sa) and phytic acid (0.1 M pa) were used on day 4, 6 and 10.

Cell proliferation of the osteoblastic cell line hFOB1.19 over a period of 10 days on the surface of set cements revealed cell numbers in the range of the control (cement with no retarding additive, set as 100% at day 4) or above and only those made with citric acid and sulfuric acid as retarder had a large decrease to approx. 27–45% in cell number after 10 days (Fig. 1A). Additionally, cell proliferation assayed by WST-test (Fig. 1B) showed lower cell activity for samples containing citric acid or sulfuric acid compared to the reference without setting retardant. However, more than three-times higher metabolic activity per cell could be reached by the use of sodium pyrophosphate or phytic acid (Fig. 1C).

**Figure 2:**
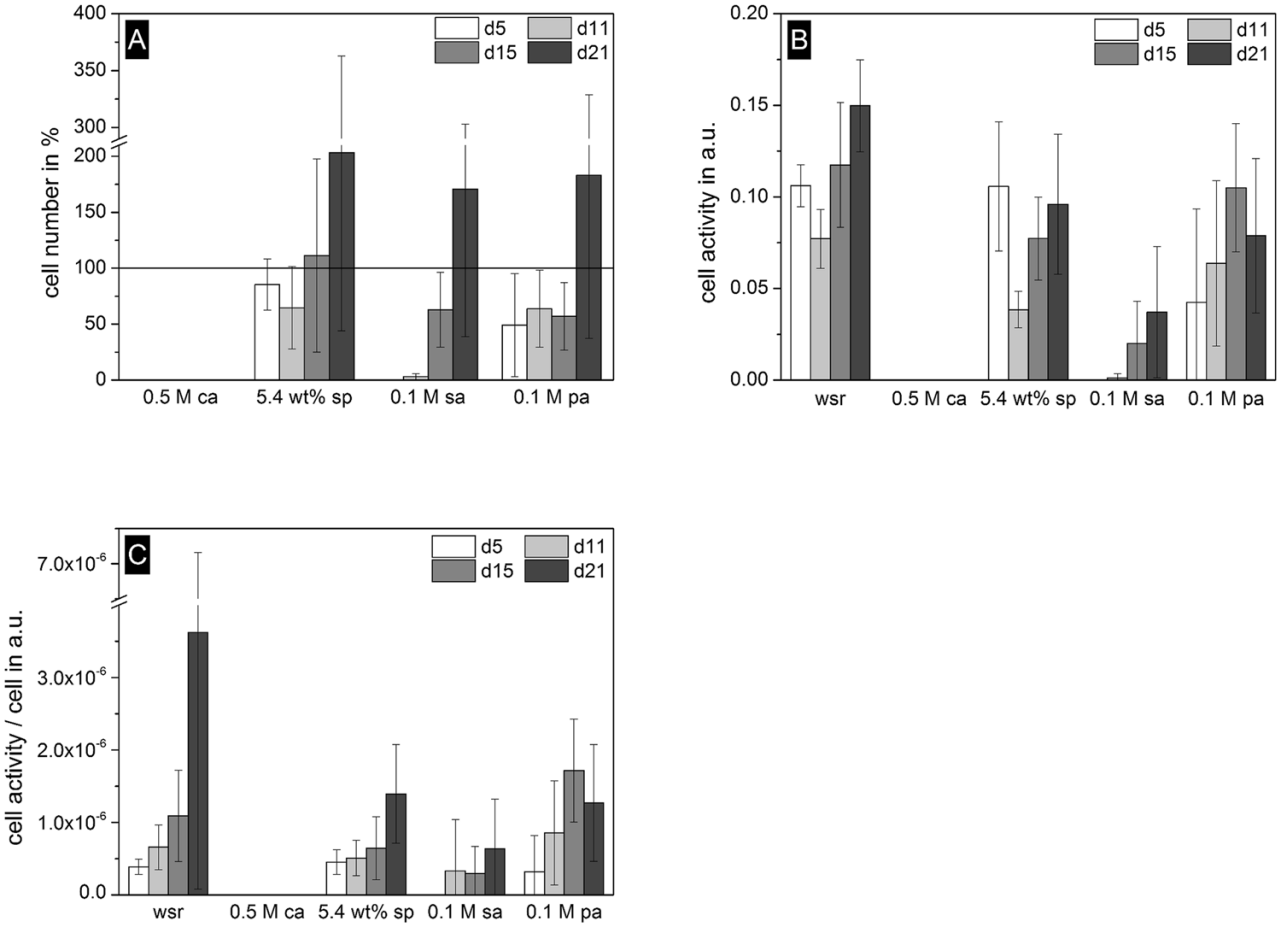
Cell number of RAW 264.7 osteoclasts with regard to cement without setting retardant (solid line at 100%) (**A**), Cell activity (**B**) and cell activity per cell (**C**). Samples without setting retardant (wsr) and retardants citric acid (0.5 M ca), sodium pyrophosphate (5.4 wt% sp), sulfuric acid (0.1 M sa) and phytic acid (0.1 M pa) were cultured over 21 days.

Additionally, cements were seeded with the macrophage cell line RAW 264.7, which were differentiated into osteoclasts after addition of 50 ng/mL RANKL. Surprisingly, cement surfaces prepared with citric acid as setting retardant fully inhibited osteoclast growth during the whole course of the experiment (Fig. 2A). The cell number of the other retarders was comparable to the reference and increased during culture of 21 days. Cell activity measured by WST-1 test is shown in Fig. 2B. Due to the lack of cells, samples with citric acid had no cell activity. Sulfuric acid had very low activity, which increased during culture period. Cements formed with sodium pyrophosphate and phytic acid were overwhelmingly comparable to the reference, even though the highest activity of the reference on day 21 could not be reached by any other composition (Fig. 2C).

**Figure 3:**
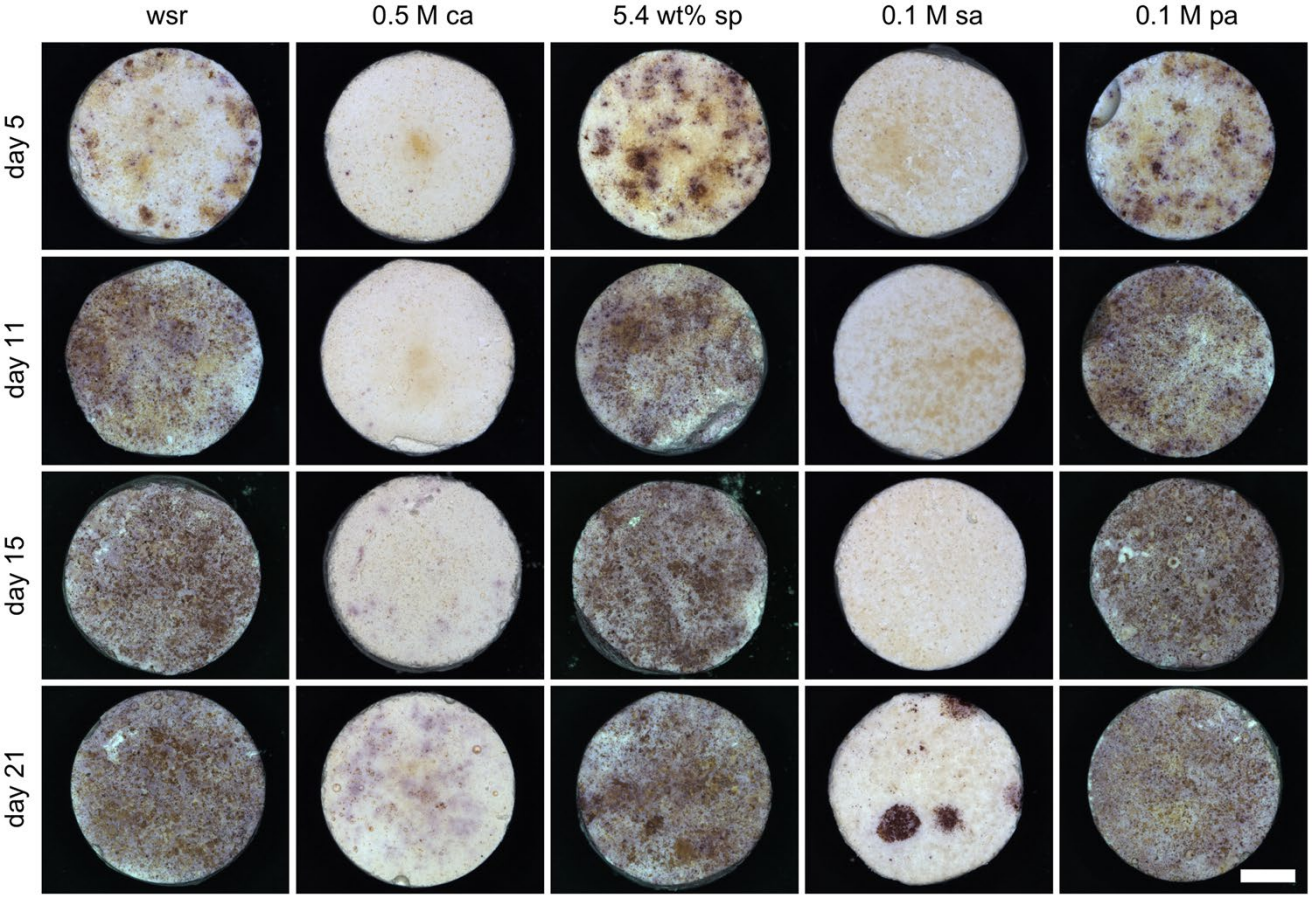
TRAP staining of RANKL treated RAW 264.7 cells. Differentiation can be seen for different setting retardants citric acid (0.5 M ca), sodium pyrophosphate (5.4 wt% sp), sulfuric acid (0.1 M sa) and phytic acid (0.1 M pa) as well as cement without setting retardant (wsr) over 21 days. Scale bar corresponds to 1.5 mm.

Differentiation of RAW macrophages into multinucleated osteoclasts by addition of RANKL was verified by TRAP staining (Fig. 3). Samples without RANKL addition served as control and were cultured over 21 days resulting in no staining for any cement composition (data not shown). Cement surfaces prepared with citric acid or sulfuric acid as retarder showed hardly any staining and only at day 21, on both surfaces little TRAP staining could be detected. In contrast, cement surfaces prepared with sodium pyrophosphate or phytic acid retarder exhibited continuously increasing TRAP staining during the culture period which was at least comparable or even more than the reference. After 15–21 days, these surfaces were confluently grown with cells.

**Figure 4:**
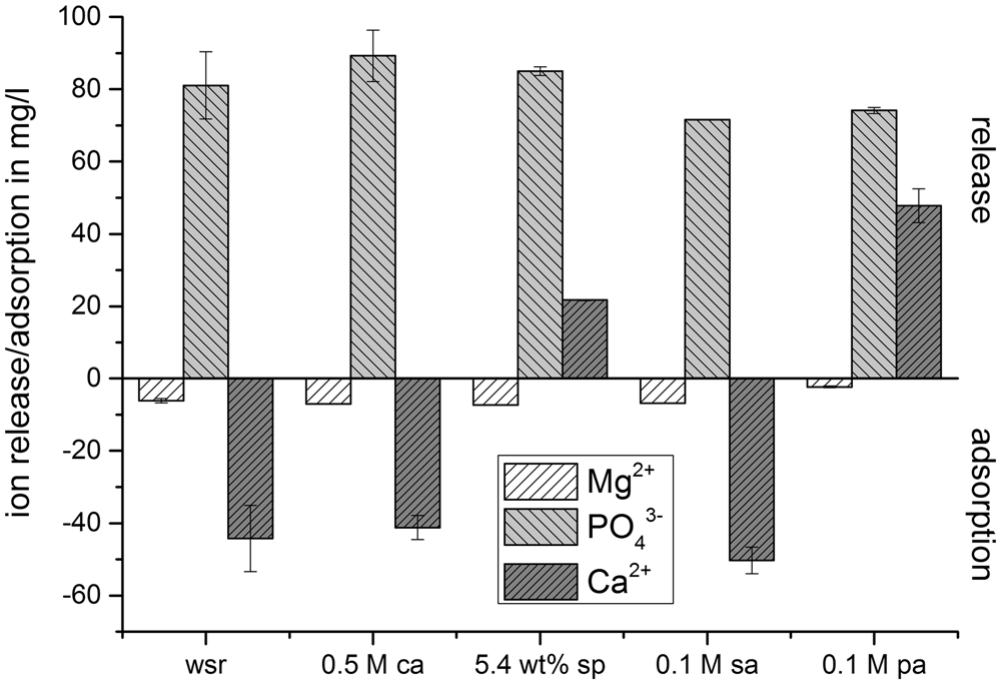
Ion concentrations after immersion of different cement samples in DMEM cell culture medium for 24 h at 37 °C in relation to fresh medium. Samples were prepared without setting retardant (wsr) and retardants citric acid (0.5 M ca), sodium pyrophosphate (5.4 wt% sp), sulfuric acid (0.1 M sa) and phytic acid (0.1 M pa).

The solubility of the preset cements was tested using DMEM cell culture medium (Fig. 4) for up to 24 h. All cements showed an adsorption of magnesium ions (2.3–7.3 mg/l) from the medium and a high release of phosphate in the range of 71–89 mg/l. Strong differences were observed regarding calcium ions, where only cements formed with sodium pyrophosphate and phytic acid increased the Ca^2+^ concentration of the medium (21–47 mg/l), whereas all other cements adsorbed calcium ions.

**Figure 5:**
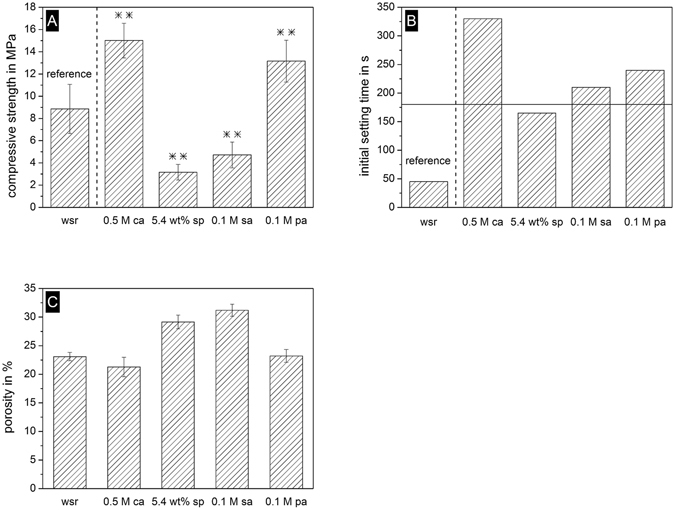
(**A**) Compressive strength of brushite with four different setting retardants. Cement without setting retardant served as reference. Samples marked with ** were highly significant (p < 0.001) with regard to the reference, (**B**) initial setting times according to Gillmore needle test (boundary for the initial setting time of 3 min is marked) and (**C**) porosity of set cements. Setting retardants citric acid (0.5 M ca), sodium pyrophosphate (5.4 wt% sp), sulfuric acid (0.1 M sa) and phytic acid (0.1 M pa) were compared to the reference without setting retardant (wsr).

Regarding the influence of setting retardants on the mechanical cement properties (Fig. 5A), the highest compressive strength was found for samples set with citric acid (15 MPa) and phytic acid (13 MPa), with both being significantly higher than the reference. In contrast, the use of sodium pyrophosphate and sulfuric acid weakened the cement matrix and compressive strength was significantly lower compared to the reference without setting retardant. Initial setting time was evaluated by the Gillmore needle test (Fig. 5B) indicating a setting time of less than one minute in the absence of any retarder. While sodium pyrophosphate, sulfuric acid and phytic acid increased the setting time to approx. 3–4 min, citric acid had a much stronger influence as retarder and prolonged cement solidification to 5.5 min. Porosity of set cements (Fig. 5C) was found to be in the range 21–23% for the reference as well as citric acid/phytic acid retarder, whereas higher values (29–31%) were obtained for sodium pyrophosphate and sulfuric acid.

**Figure 6:**
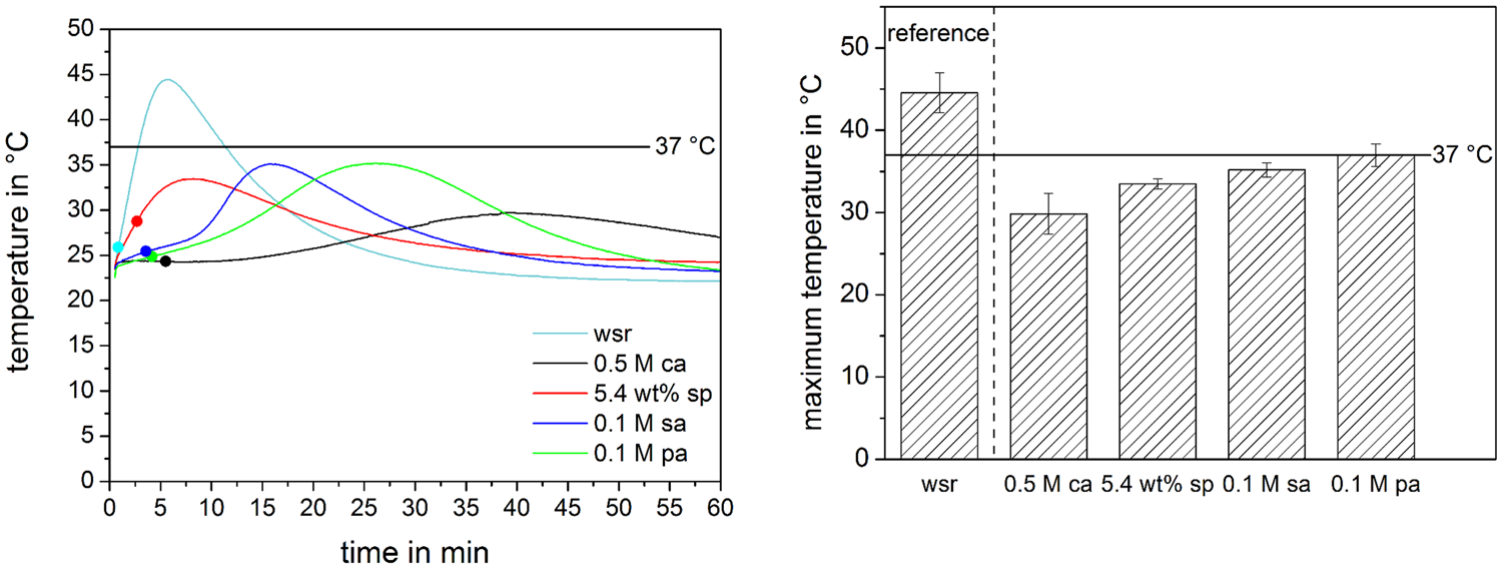
Temperature profile between 30 s and 60 min (left) and maximum temperature reached during setting (right). Samples with setting retardants citric acid (0.5 M ca), sodium pyrophosphate (5.4 wt% sp), sulfuric acid (0.1 M sa) and phytic acid (0.1 M pa) as well as a reference without setting retardant (wsr) ware examined. Dots mark the initial setting time determined by Gillmore needle test.

**Figure 7:**
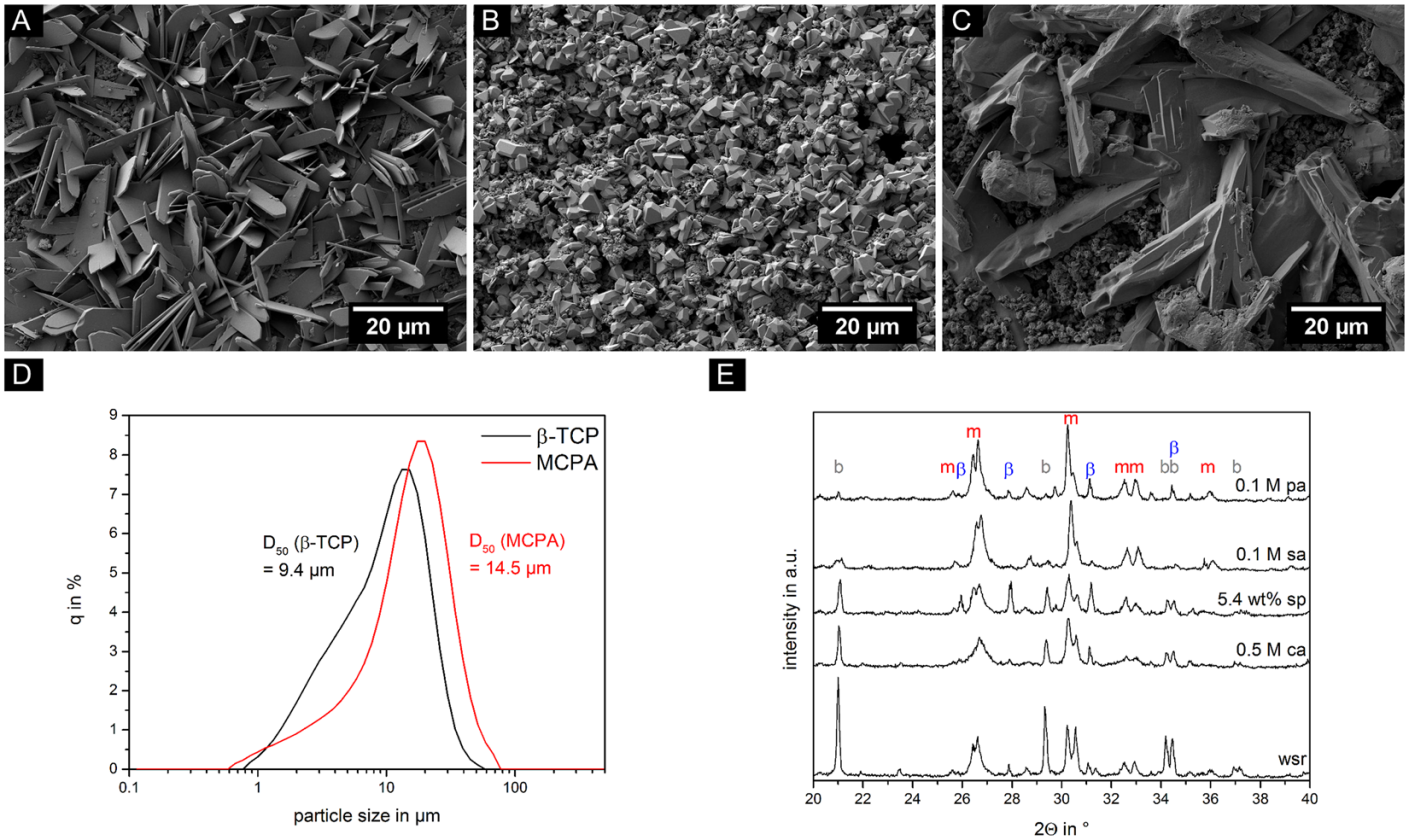
SEM images of three different types of morphology taken at 3 kV and 2000-fold magnification. Images show plates (**A**), plate-like crystals (**B**) and needles (**C**). The cements with 0.1 M sulfuric acid (**A**), no setting retarder (**B**) and 0.5 M citric acid (**C**) served as examples. The particle size distribution of the reactant powders β-TCP and MCPA are shown (**D**). Both had a monomodal particle size distribution between 1 µm and 100 µm. Analysis of phase composition via XRD (**E**) exhibited crystalline structures for brushite (b), monetite (m) and the reactant powder β-TCP (β).

Figure 6 shows the temperature evolution during cement setting over a period of 60 min and a dot marks the initial setting time measured with Gillmore needle test. As could be already seen at the Gillmore needle test, cement samples set without retardant had the earliest exothermic reaction and highest maximum temperature (45 °C). The maximum peak during setting reaction is shifted to later time points when retarders were employed and also the temperatures achieved during setting decreased by the addition of retarders below 37 °C. Morphological analysis using SEM revealed three types of crystals: plates (Fig. 7A), needles (Fig. 7C) and paving stone like structures (Fig. 7B), differing in terms of aspect ratio (two-dimensional) and three-dimensional expansion. While cements formed with sodium pyrophosphate and citric acid showed a predominantly needle like structure with a crystal size of 5–10 µm, sulfuric acid as well as phytic acid as retarders led to the formation of plate-like crystals of <5 µm size. A more compact morphology was observed in the absence of any retarder (Fig. 7B). The size distribution of the cement raw materials (Fig. 7D) was monomodal with a medium size of ß-TCP (9.4 µm) and MCPA (14.5 µm).

Phase analysis performed with XRD (Fig. 7E) exhibited three different phases: brushite (b), the anhydride monetite (m) and the cement raw powder β-TCP (β). The second cement component MCPA was not found in crystalline form after setting. When phytic acid or sulfuric acid were used as retarders, monetite was the predominant phase formed during setting, whereas for all other retardants the formation of mixtures of both brushite and monetite was observed, even though there were residues of β-TCP in all compositions.

## Discussion

Dicalcium phosphate crystals are formed during cement setting by a comparatively fast dissolution/precipitation process^5^. The reactants β-TCP and MCPA dissolve in the liquid phase, whereas brushite or monetite precipitates at a pH-value less than 4.2^5^. However, the setting time of dicalcium phosphate cements without any retardant of less than one minute is very short. An extension of the setting time to a clinically applicable time frame requires the use of setting retarders, such as pyrophosphates, sulfates or citrates^24^. These compounds delay the crystal growth rate of the setting product by adsorbing at the active growth sites of dicalcium phosphate crystal faces^34^, leading to both a longer setting time (Fig. 5B) and a decrease in the maximum setting temperature at later time points (Fig. 6B). Among these retarders, citric acid has been proven to simultaneously retard setting and attain excellent material properties such as high compressive strength^31^ and good injectability^30^. This was confirmed in the present study demonstrating the highest cement strength and longest setting times compared with all other additives (Fig. 5). The only other compound leading to nearly similar cement characteristics was phytic acid, which is known to form Ca^2+^/Mg^2+^ chelates with compounds such as α-tricalcium phosphate, ß-tricalcium phosphate or trimagnesium phosphate^35–37^ and which acts as a regulator of brushite and hydroxyapatite crystal growth^38^.

Although for all cements a constant powder to liquid ratio was used, porosity as a strength determining parameter^39, 40^ was found to vary in the range from 21–31% (Fig. 5C). These porosity differences likely correlate with strength variation as those cements with the lowest porosity (citric acid, phytic acid, no retarder) had the highest strength, whereas the Ca^2+^ chelating capacity of citric acid and phytic acid may have further contributed to the mechanical performance. In addition, the observed variation in brushite crystal morphology may be a regulating parameter for cement strength as it is also known for hydroxyapatite forming cements^41^. Examination of surface morphology of the different setting retardants via SEM revealed three types of crystal shapes. Plates and needles could be detected due to a non-centrosymmetric monoclinic crystallization structure as reported elsewhere^42, 43^. Furthermore, paving stone like structures were found having a more compact structure with a lower aspect ratio compared to plates and needles. In most cases, the morphology of cement samples was a mixture of these three types since the complex kinetics influenced by numerous factors may play a decisive role in crystallization leading to a variety of crystal morphologies. In terms of the mechanical cement properties, the predominant needle like character of brushite crystals formed by the addition of citric acid are thought to result in a better entanglement of crystals compared to the compact morphology of brushite crystals without setting retarder (Fig. 7).

Despite of the excellent cement material properties, recent studies indicated a reduced cell attachment on cement surfaces^33^ and a strongly delayed degradation *in vivo* ^17^ for citric acid modified brushite cements. Especially the latter is surprising, since the authors did not observe any phase changes during 10 months of orthotopic implantation for low porosity brushite cements. This anomalous behavior may have two reasons: firstly, the calcium citrate complexes formed in the cement may generally decrease the solubility of the cement and/or secondly, the calcium citrate complexes are not only affecting the proliferation/activity of osteoblasts but also those of bone resorbing osteoclasts. Regarding the cement solubility, cements formed with citric acid showed a similar dissolution behavior like cements with sulfuric acid or without setting retarder (Fig. 4). The release of phosphate in conjunction with the adsorption of Ca^2+^ and Mg^2+^ is related to the well know tendency of brushite cements to convert into calcium phosphate phases with a higher Ca: P ratio^44–47^. Surprisingly, sodium pyrophosphate and phytic acid behaved differently and expressed both Ca^2+^ and phosphate ions. However, since the release was measured only at an early time point, an absence or a retarded phase transformation of brushite into lower soluble phases in a long term implantation regime is speculative.

The biological part of our study confirmed recent results of Jamshidi *et al*.^33^ regarding the low cytocompatibility of brushite cements formed with citric acid retarder. The human fetal osteoblast cell line hFOB used here is likely more suitable for bone models than tumor derived MG63 exhibiting numerous karyotypic alterations^48^. Cell number of hFOB was measured via cell counting on day 4, 6 and 10 (Fig. 1) showing that most cement samples were in the range of the retardant free reference with a slight tendency to higher cell numbers for sodium pyrophosphate modification. Citric acid, in contrast, had decreasing cell numbers, especially on day 10. Cell activity measurements confirmed the tendency suggested for cell number and activity on citric acid and sulfuric acid modified cements was even lower than for the reference tracing back to a reduced cell metabolism. In contrast, the addition of sodium pyrophosphate or phytic acid enhanced cell activity up to three times indicating a metabolism activating influence on osteoblasts.

Complementary to osteoblast cell tests, osteoclast precursor cells RAW264.7 were cultured on cement surfaces over 21 days (Fig. 2). This cell line is an established model to study bioceramic dissolution for both sintered hydroxyapatite or tricalcium phosphate ceramics^49^ as well as for low temperature cements^50^. The effect of citric acid on osteoclasts was even more striking than for osteoblastic cells showing practically no cell growth or cell activity during the whole culture period. Sulfuric acid also showed very low cell number and cell activity in the first eleven days but increased until day 21. Sodium pyrophosphate and phytic acid were almost comparable to the reference and showed no inhibiting effect on osteoclast proliferation and activity. To prove differentiation of precursor cells into multinucleated osteoclasts by supplementation of RANKL to the medium, staining with TRAP was performed (Fig. 3). On surfaces with citric acid and sulfuric acid, no staining could be found which is in accordance with cell number and cell activity. In contrast, on samples with sodium pyrophosphate and phytic acid all cells differentiated within 15 days and stayed vital until day 21. The results clearly support the hypothesis that citric acid used as a retarder in brushite cements alters the degradation ability of the cement *in vivo*, most likely because of a cytotoxic effect on osteoclastic cells. However, brushite cements in the current study contained a relatively high concentration of citric acid (0.5 M), since this concentration provided the maximum effect on cement material properties. Previous experiments with other citrate modified cements were performed with 10 times lower concentrations of 0.05 M and showed medium activity of osteoclasts. This demonstrates that citrate ions are not generally cytotoxic for osteoclasts and hence this may open the possibility for further cement optimization regarding cytocompatibility and material properties. The mechanism by which citrate ions inhibit osteoclast formation and activity is however still unclear. A recent study by Drager *et al*.^51^ has shown a strong effect of iron chelators on osteoclast function, as iron is needed for mitochondrial biogenesis during osteoclastic differentiation. Since citrate has also the capacity to form iron-chelates^52^, the formation of such complexes from dissolved calcium citrate from the cement might have provoked iron chelation with the observed inhibition of osteoclast formation. Although also phytic acid is a strong chelating agent for iron^53^, we assume, that the calcium-phytate complexes formed during cement setting are stable under *in vitro* neutral pH conditions as demonstrated by Gosselin *et al*.^54^ and Grynspan *et al*.^55^ in contrast to calcium citrate which dissolves in this environment^56^. It is then likely that for phytic acid as setting retarder, the free iron concentration is maintained within the cell culture (and hence osteoclast activity), which is not the case for citric acid retarder due to the solubility of calcium citrate.

## Conclusion

An *in vitro* cytocompatibility test with both osteoblastic (hFOB1.19) and osteoclastic (RAW264.7) cell lines revealed a very low cell number and activity for citric acid as a common dicalcium phosphate cement setting retardant. To overcome this drawback, we analyzed the retarders sodium pyrophosphate, sulfuric acid and phytic acid, whereas the best *in vitro* performance was achieved with phytic acid with a high cell proliferation and cell activity. The mechanical properties were investigated with a compression test, showing that cements with sodium pyrophosphate (CS = 3.2 ± 0.7 MPa) and sulfuric acid (CS = 4.7 ± 1.2 MPa) were mechanically much weaker than cements formed with phytic acid (CS = 13.2 ± 1.9 MPa) or citric acid (CS = 15.0 ± 1.6 MPa). Altogether, phytic acid as a novel setting retardant for dicalcium phosphate cements seemed to be promising in terms of material properties as well as biological behavior. Nevertheless, further knowledge has to be gained by *in vivo* tests, since complex organisms always have a different behavior compared to mono-cultures.

## Materials and Methods

### Sample preparation

β-TCP was made of an equimolar mixture of CaHPO_4_ and CaCO_3_ (both Merck, Darmstadt, Germany) by sintering for 14 h at 1400 °C followed by 6 h at 1000 °C. Crushing with mortar and pestle followed by grinding for 10 min in a planetary ball mill (PM400, Retsch, Germany) resulted in a medium particle size of 9.4 µm. Monocalcium phosphate anhydrate (MCPA) was used as phosphate source. MCPA (LOT: MV3272, Budenheim) was mixed in a stoichiometric ratio of 0.755:1 with β-TCP within a coffee mill (Privileg, type: PCML2012, Quelle GmbH, Fürth, Germany). The setting retardants citric acid monohydrate (Sigma-Aldrich, LOT: SZBB2010V), sulfuric acid (Merck, LOT: 318 K19691813) and phytic acid (Sigma-Aldrich, LOT: BCBH8997V) were added to the liquid phase, whereas tetra-sodium pyrophosphate decahydrate (Merck, LOT: F1061091 139) was added to the powder. The concentrations were 0.5 mol/L citric acid (0.5 M ca), 5.4 wt% sodium pyrophosphate (5.4 wt% sp), 0.1 mol/L sulfuric acid (0.1 M sa) and 0.1 mol/L phytic acid (0.1 M pa). All samples had a powder to liquid ratio of 3 g/mL and were produced in silicon molds. Samples were set for 1 h at 37 °C and 100% humidity prior to testing.

### Characterization

Particle sizes of raw powders were measured with a laser diffractometer (Horiba LA 300 Wet, Horiba, Japan) in ethanol. Characterization of the cement morphology was performed by cross beam scanning electron microscopy (SEM; CB 340, Zeiss, Germany) where the samples were sputtered with 4 nm platinum prior to imaging. The SEM operated at 3 kV with a 2000-fold magnification. For mechanical testing, cuboid samples of 6 mm × 6 mm × 12 mm (n = 12) were prepared and stored in water at 37 °C for 24 h. The mechanical testing device (Zwick GmbH & Co.KG, Ulm, Germany) was operated in compression mode with a 10 kN load cell at a testing speed of 1 mm/min. The solubility of preset cement samples was tested by immersion in 10 ml DMEM cell culture medium for 24 h at 37 °C. Ion concentrations in the immersion liquids were quantified by inductively coupled plasma mass spectrometry (ICP-MS; Varian, Darmstadt, Germany) against standard solutions of 5 and 10 mg/mL (Merck, Darmstadt, Germany). To measure the initial setting time, a Gillmore needle test was performed within a chamber at 37 °C and 100% humidity. Exothermic behavior of isolated samples of 1.5 g cement (n = 5) within 60 min was recorded by a thermometer (Data-Logger Thermometer 306, Conrad Electronic GmbH, Hirschau, Germany). Statistical analysis was calculated with ANOVA using the program SigmaPlot 12.5 (Systat Software, Inc., 2011). The porosity of the cement samples (6 mm × 6 mm × 12 mm) was measured according to Unosson *et al*.^57^, by solvent resaturation (n = 12). Therefore, the weight of water saturated samples was recorded followed by drying under vacuum at room temperature. The porosity Φ in percent was calculated according to Eq. (1), where *m*_*sat*_ and *m*_*dry*_ are the weight of saturated and dry samples, respectively, *ρ*_*w*_ is the density of water and *V* is the apparent volume of the sample.

1 $${\rm{\Phi }}=(\frac{({m}_{sat}-{m}_{dry})/{\rho }_{w}}{V})\ast 100$$

Phase analysis was performed with x-ray diffraction (XRD; Siemens D5005, Bruker AXS, Karlsruhe, Germany) operating with Cu-K_α_ radiation (λ = 0.15418 nm) at a voltage of 40 kV and a current of 40 mA. Samples were scanned in a range of 2Θ = 20°–40° with a scan rate of 1.5 s/step and a step size of 0.02°. Phases were identified with the help of ICDD (International Centre for Diffraction Data) database for brushite (PDF-No. 09–0077), β-TCP (PDF-No. 09–0169), MCPA (PDF-No. 09–0390) and monetite (PDF-No. 09–0080).

### Biological testing

To exclude any influence of different cement pHs on the biological results, sample discs with a diameter of 15 mm and a height of 2 mm were washed with autoclaved ultrapure water and phosphate buffered saline (PBS) until a physiological pH-value of the washing solution was achieved, followed by twice an immersion in 70% ethanol for 30 min. Cells of the human fetal osteoblast cell line hFOB 1.19 were cultured in Dulbecco’s Modified Eagle’s Medium (DMEM/F12, Gibco, Cat. No.: 313311–028) supplemented with 1% Penicillin-Streptomycin (Gibco, Cat. No.: 15140–122), 0.3 g/L Geneticin G-418 Sulphate (Gibco, Cat. No.: 11811–064) and 10% fetal calf serum (FCS, Gibco, Cat. No.: 10270–106). Cement samples (n = 4) and polystyrene plates serving as reference were placed in 24-well plates and seeded with a cell density on cement surfaces of 10^5^ cells/well and 5 · 10^4^ cells/well on polystyrene. The seeded samples were incubated at 34 °C and 5% CO_2_ and cell activity and cell number were evaluated at day 4, 6 and 10. Samples were incubated for 30 min with WST-1 reagent (dilution of 1:10) and the cell activity was measured twice with a spectrometer (Tecan SpectraFluor Plus, Tecan, Maennedorf, Switzerland). Cell number was evaluated by a cell counter (Casy, Roche innovates AG, Bielefeld, Germany) after detaching cells from the surface by 12 min incubation with Accutase (PAA, Cat. No.: L11–007). Cell counting was performed in isotonic liquid with a dilution of 1:100. Furthermore, cell tests with the murine macrophage cell line RAW 264.7 were performed to investigate the influence of retardant addition on activity of bone degrading cells. RAW cells were used up to passage 18 and were cultured in Dulbecco’s Modified Eagle’s Medium (DMEM; ATCC), supplemented with 1% Penicillin-Streptomycin (Gibco, Cat. No.: 15140–122) and 10% FCS (Gibco, Cat. No.: 10270–106). Therefore, samples (n = 4) with a diameter of 5 mm and a height of 2 mm were placed in 96-well plates, seeded with 6.4 · 10^3^ cells/well and incubated at 37 °C and 5% CO_2_. After 24 h of culture medium was supplemented with 50 ng/mL receptor activator of the nuclear factor κB ligand (RANKL) to induce differentiation to multinucleated osteoclasts and culture medium was changed every 2–3 days. Osteoclast differentiation was evaluated by tartrate resistant acid phosphatase (TRAP) staining after 5, 11, 15 and 21 days by using a commercial TRAP-staining kit (Sigma, Cat. No. 387). Moreover, cell number of osteoclasts was determined using Auto T4 cell counter (Nexcellom Bioscience, Lawrence, Massachusetts, USA) after detaching cells from cement surface by incubation with Accutase (PAA, Cat. No.: L11–007) for 45 min. Similar to hFOB cell tests, activity of osteoclasts was measured by WST-1 reagent also over 21 days.
